# Supplementary material for: The MacqD deep-learning-based model for automatic detection of socially housed laboratory macaques
Source: Sci Rep. 2025 Apr 7;15:11883. doi: 10.1038/s41598-025-95180-x (PMC11977019; doi:10.1038/s41598-025-95180-x)
Supplement: Supplementary file 1 — Supplementary Information. [file 41598_2025_95180_MOESM1_ESM.pdf]

Supplementary materials

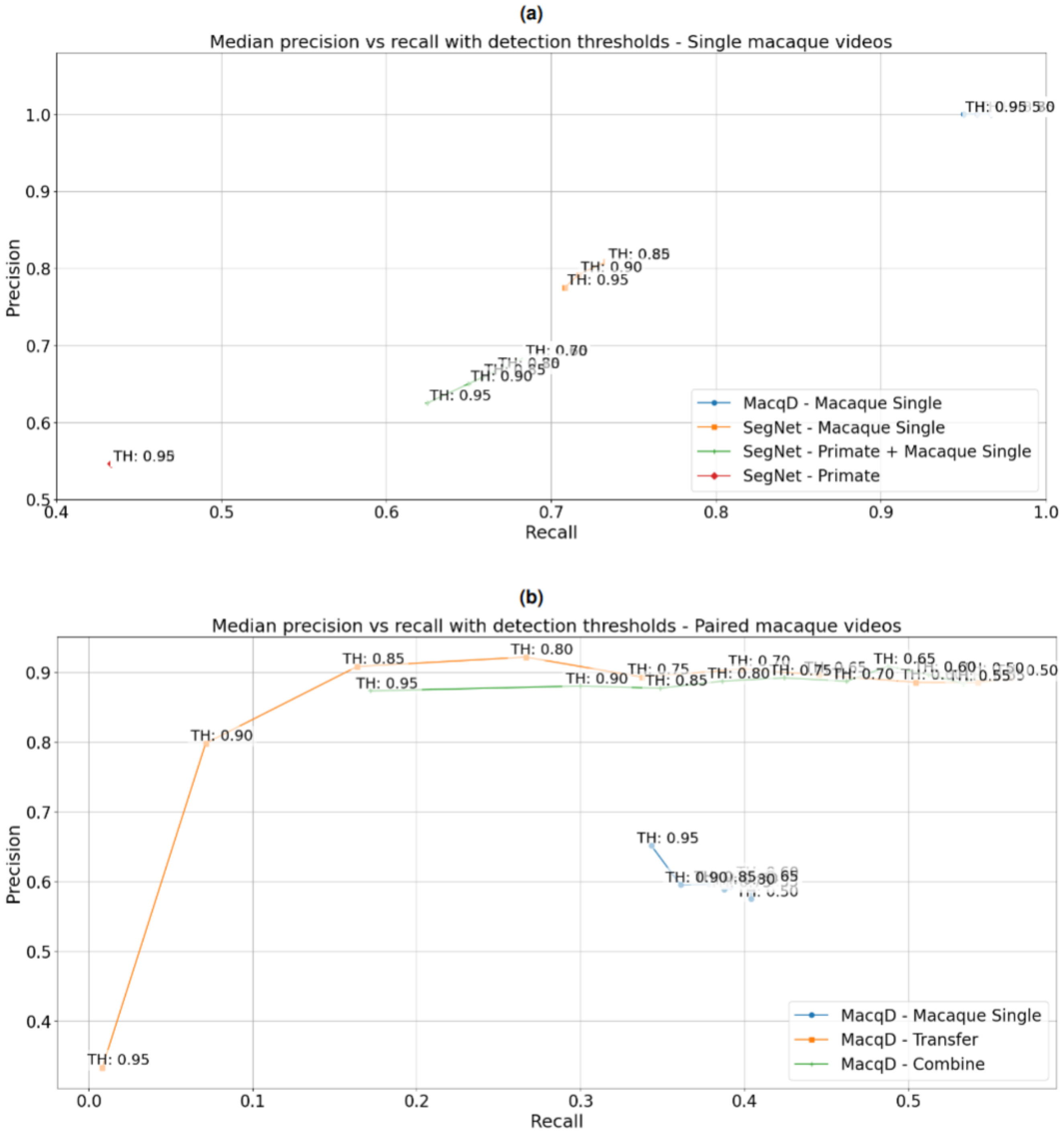

Figure S1. (**a**) Median precision vs. recall across various confidence score thresholds on the validation dataset with a single macaque in the focal cage. Optimal thresholds are: SegNet - Primate (0.95), SegNet - Primate + Macaque Single (0.7), SegNet - Macaque Single (0.85), and MacqD - Macaque Single (0.8). (**b**) Median precision vs. recall across various confidence score thresholds on the validation dataset with a pair of macaques in the focal cage. The optimal threshold for MacqD - Macaque Single, MacqD - Macaque Curriculum, and MacqD - Macaque Combine is 0.5.

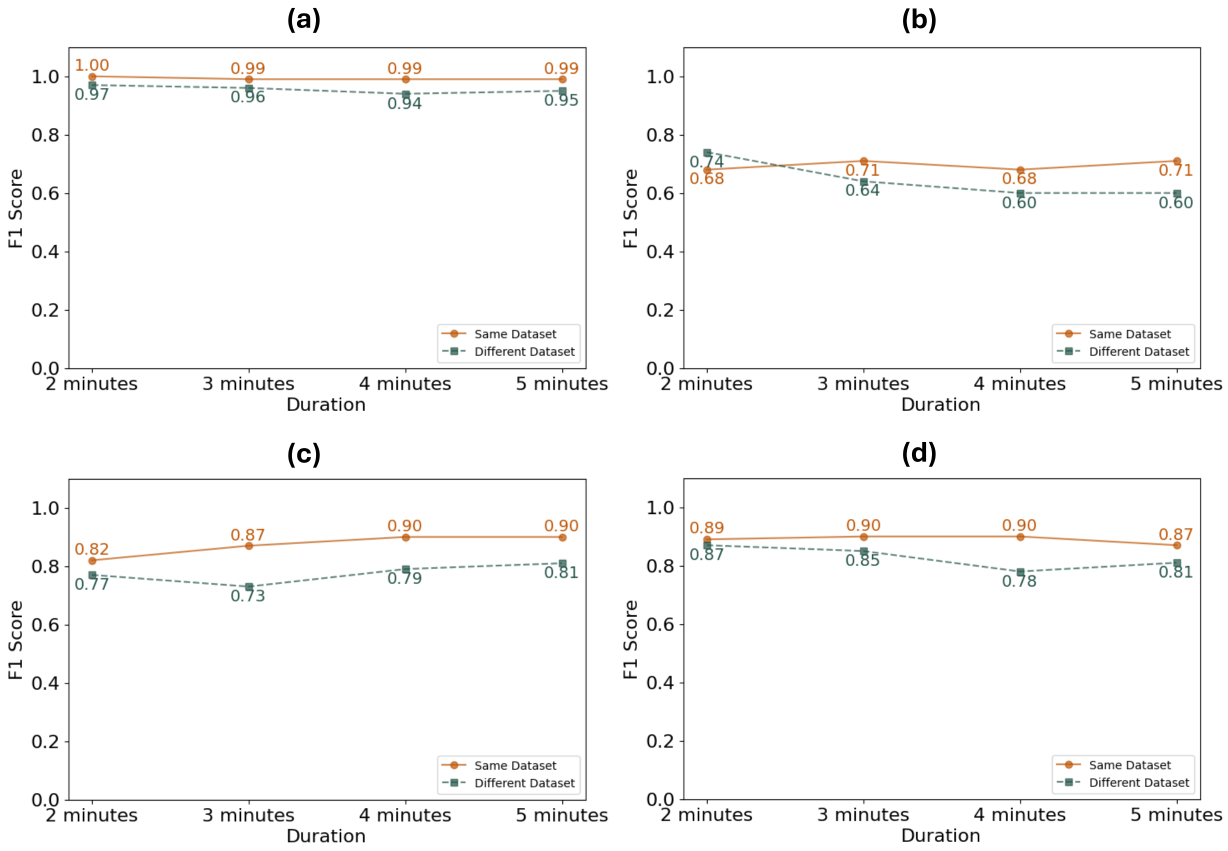

Figure S2. F1 scores of MacqD tested with different video durations (2–5 minutes). (**a**) MacqD - Macaque Single tested on a dataset featuring a single macaque in the focal cage. (**b**) MacqD - Macaque Single tested on a dataset featuring paired macaques in the focal cage. (**c**) MacqD - Macaque Curriculum tested on a dataset featuring paired macaques in the focal cage. (**d**) MacqD - Macaque Combine tested on a dataset featuring paired macaques in the focal cage. "Same dataset" refers to individuals that were included in the training dataset, while "Different dataset" refers to individuals that were not used during training. The F1 scores at 5 minutes correspond to the values reported in the Results section.

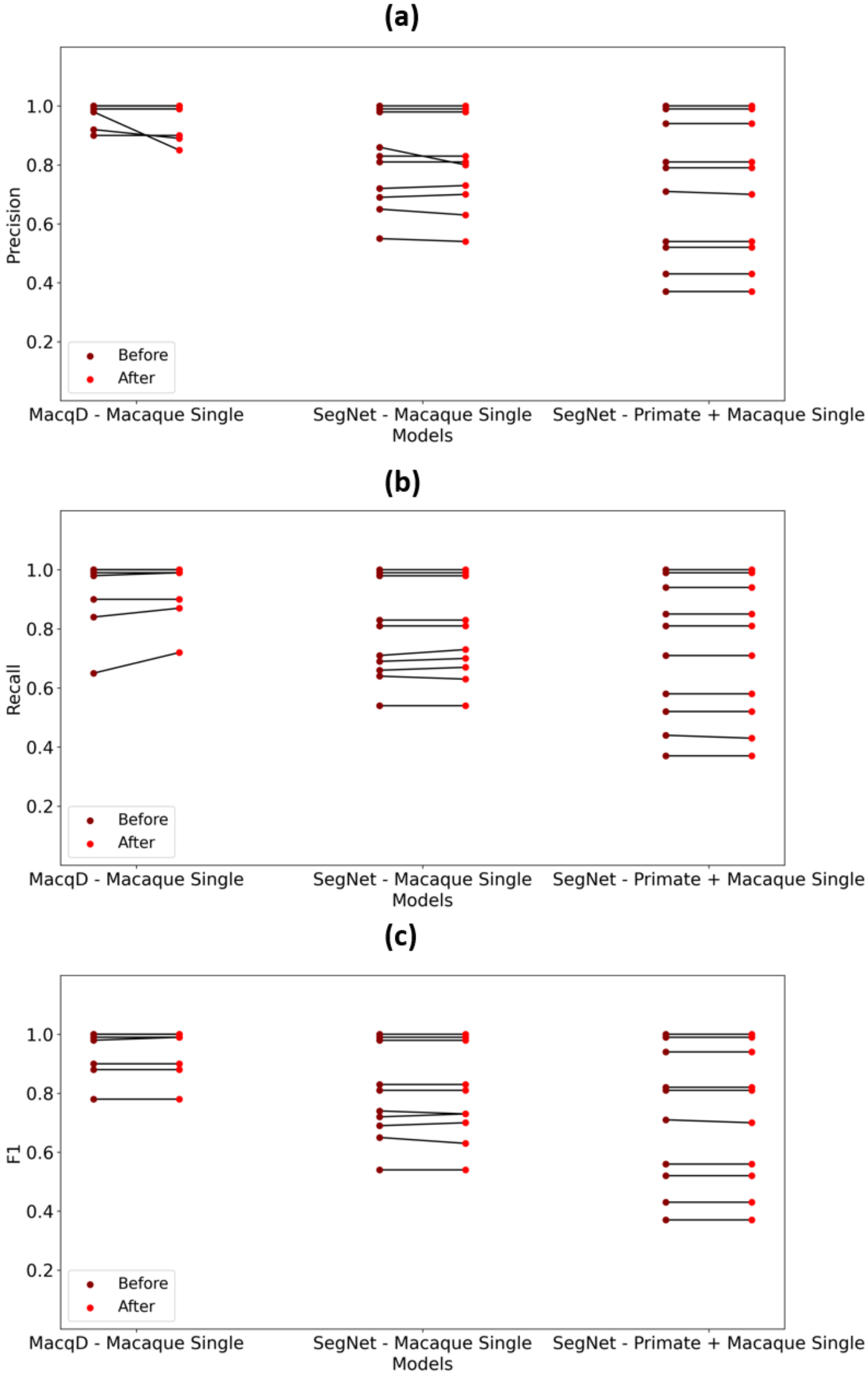

Figure S3. Visual representation of model predictions on frames featuring single macaques in the focal cage, which are the same individuals from the training dataset (Experiment 1, 'Same' dataset), before and after applying the tracking algorithm. (**a**) Precision; (**b**) Recall; (**c**) F1 Score.

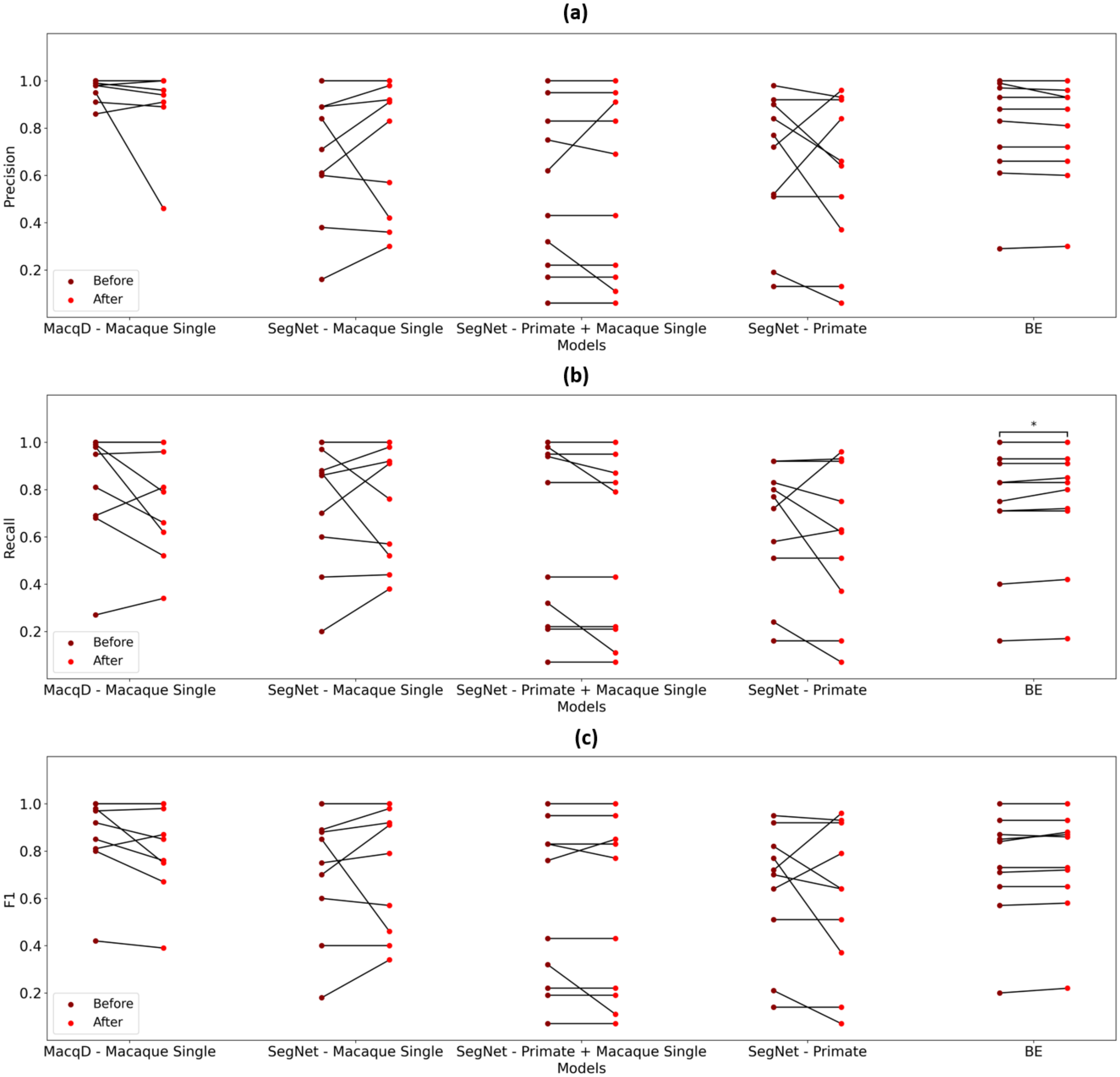

Figure S4. Visual representation of model predictions on frames featuring single macaques in the focal cage, which are the different individuals from the training dataset (Experiment 1, 'Different' dataset), before and after applying the tracking algorithm. (**a**) Precision; (**b**) Recall; (**c**) F1 Score. Bar indicates significant differences before and after applying the tracking algorithm (Wilcoxon test: $p < 0.05$).

Model performance

Model	Dataset	Before tracking algorithm implementation			After tracking algorithm implementation		
		Precision	Recall	F1	Precision	Recall	F1
MacqD - Macaque Single	'Same'	0.99	0.99	0.99	0.99	0.99	0.99
SegNet - Macaque Single		0.82	0.76	0.78	0.81	0.77	0.77
SegNet - Primate + Macaque Single		0.75	0.76	0.76	0.75	0.76	0.76
MacqD - Macaque Single	'Different'	0.99	0.97	0.95	0.96	0.8	0.86
SegNet - Macaque Single		0.78	0.87	0.8	0.87	0.84	0.85
SegNet - Primate + Macaque Single		0.53	0.63	0.60	0.56	0.61	0.6
SegNet - Primate		0.75	0.75	0.71	0.65	0.53	0.64
BE		0.86	0.79	0.78	0.85	0.82	0.80

Wilcoxon test

Model	Dataset	N	DOF	Metric of comparison	T^+	p-value
MacqD - Macaque Single	'Same'	10	1	Precision	0	0.180
				Recall	0	0.109
				F1	0	0.317
SegNet - Macaque Single				Precision	4	0.336
				Recall	2	0.257
				F1	4	0.705
SegNet - Primate + Macaque Single				Precision	0	0.317
				Recall	0	0.317
				F1	0	0.317
MacqD - Macaque Single	'Different'			Precision	7.5	0.270
				Recall	6	0.176
				F1	4	0.091
SegNet - Macaque Single				Precision	11.5	0.362
				Recall	16	0.779
				F1	8	0.310
SegNet - Primate + Macaque Single				Precision	3	1
				Recall	0	0.109
				F1	2	0.593
SegNet - Primate				Precision	10	0.499
				Recall	9	0.398
				F1	10	0.499
BE				Precision	2	0.131
				Recall	0	**0.042**
				F1	2	0.071

Table S1. Summary of model performance and statistical comparisons on frames featuring single macaques in the focal cage (Experiment 1), before and after applying the tracking algorithm. Bold number indicates a significant difference in the metric results before and after applying the tracking algorithm.

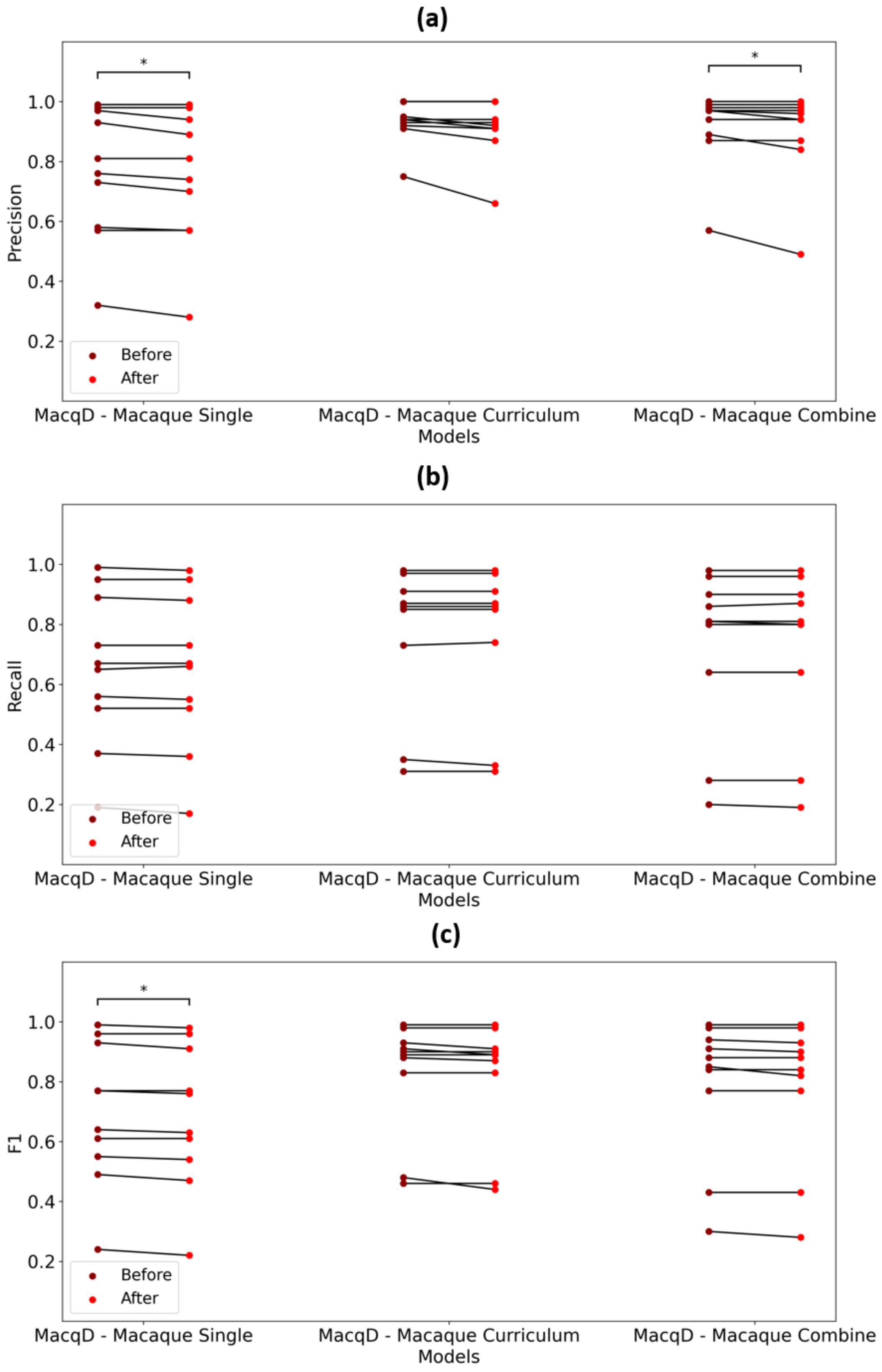

Figure S5. Visual representation of model predictions on frames featuring paired macaques in the focal cage, which are the same individuals from the training dataset (Experiment 2, ’Same’ dataset), before and after applying the tracking algorithm. (**a**) Precision; (**b**) Recall; (**c**) F1 Score. Bars indicate significant differences before and after applying the tracking algorithm (Wilcoxon test: $p < 0.05$).

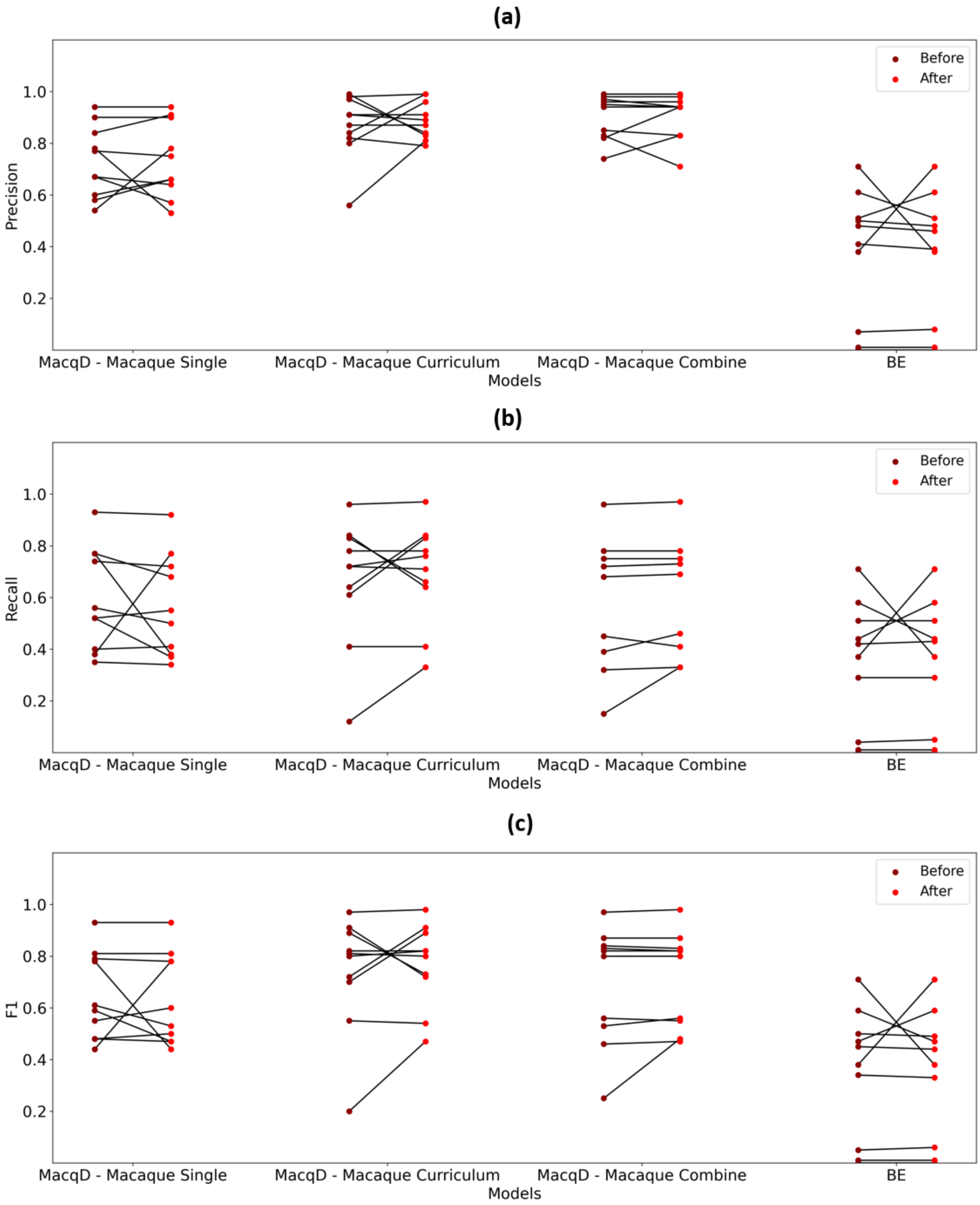

Figure S6. Visual representation of model predictions on frames featuring paired macaques in the focal cage, which are the different individuals from the training dataset (Experiment 2, 'Different' dataset), before and after applying the tracking algorithm. (**a**) Precision; (**b**) Recall; (**c**) F1 Score.

Model performance

Model	Dataset	Before tracking algorithm implementation			After tracking algorithm implementation		
		Precision	Recall	F1	Precision	Recall	F1
MacqD - Macaque Single	'Same'	0.79	0.66	0.71	0.78	0.67	0.70
MacqD - Macaque Curriculum		0.94	0.87	0.90	0.93	0.87	0.89
MacqD - Macaque Combine		0.97	0.81	0.87	0.95	0.81	0.86
MacqD - Macaque Single	'Different'	0.72	0.54	0.6	0.71	0.53	0.57
MacqD - Macaque Curriculum		0.89	0.72	0.81	0.88	0.74	0.81
MacqD - Macaque Combine		0.95	0.7	0.81	0.94	0.71	0.81
BE		0.45	0.40	0.42	0.43	0.40	0.41

Wilcoxon test

Model	Dataset	N	DOF	Metric of comparison	T^+	p-value
MacqD - Macaque Single	'Same'	10	1	Precision	0	**0.027**
				Recall	3	0.096
				F1	0	**0.016**
MacqD - Macaque Curriculum				Precision	0	0.068
				Recall	2	0.564
				F1	0	0.068
MacqD - Macaque Combine				Precision	0	**0.042**
				Recall	1	0.655
				F1	0	0.066
MacqD - Macaque Single	'Different'			Precision	17	0.889
				Recall	16	0.275
				F1	14.5	0.623
MacqD - Macaque Curriculum				Precision	9.5	0.833
				Recall	6	0.089
				F1	10.5	0.547
MacqD - Macaque Combine				Precision	16	0.779
				Recall	22	0.326
				F1	16	0.438
BE				Precision	14	0.574
				Recall	9	0.752
				F1	14	0.573

Table S2. Summary of model performance and statistical comparisons on frames featuring paired macaques in the focal cage (Experiment 2), before and after applying the tracking algorithm. Bold numbers indicate a significant difference in the metric results before and after applying the tracking algorithm.
